# The development and validation of the psychological capital questionnaire for patients with Cancer the psychological capital questionnaire

**DOI:** 10.1186/s12885-021-08960-9

**Published:** 2021-11-10

**Authors:** Chun Ying Cui, Yu Wang, Ying Zhang, Siqi Chen, Nan Jiang, Lie Wang

**Affiliations:** Department of Social Medicine, School of Public Health, China Medical University, No.77 Puhe Road, Shenyang North New Area, Shenyang, Liaoning 110122 People’s Republic of China

**Keywords:** Cancer, Psychological capital, Questionnaire, PCQ-C, Development

## Abstract

**Background:**

Studies increasingly show that positive psychological constructs affect the mental health of cancer patients. However, most scales that measure hope, resilience, optimism and self-efficacy have been developed based on general populations. The aim of our study was to develop a psychological capital (PsyCap) questionnaire for patients with cancer (PCQ-C) to gauge their mental state more accurately.

**Methods:**

The items for the scale were selected by comprehensive literature review and semi-structured interviews, and the relevant terms were screened by an expert panel. A pilot study was then conducted on 202 patients to reduce the item pool, and the reliability and validity of the scale were evaluated using 500 completed questionnaires. The test-retest reliability was then assessed using a subsample of 100 patients. Finally, the completed questionnaires of 229 patients with breast cancer were used to assess the criterion validity of the PCQ-C, including measures of depression and anxiety.

**Results:**

Item reduction and exploratory factory analysis resulted in 24 items for self-efficacy, hope, resilience and optimism, accounting for 56.72% of the variance. The Cronbach’s alpha for the scale was 0.886, and the test-retest reliability was 0.825. PsyCap showed a significant negative correlation with both depression (*r* = − 0.631, *P* < 0.01) and anxiety (*r* = − 0.601, *P* < 0.01).

**Conclusion:**

The PCQ-C can objectively evaluate PsyCap in cancer patients and exhibits good psychometric properties.

## Background

Several state-like positive psychological constructs have been identified in the fields of positive psychology and psycho-oncology [1, 2]. The most common state-like constructs are hope (persevering towards goals and when necessary, redirecting paths to reach those goals), resilience (withstanding problems and adversity, and rebounding), optimism (making a positive attribution about reaching goals) and self-efficacy (having confidence to take on and succeed at challenging tasks) [3, 4], in addition to wisdom, gratitude, courage, well-being and forgiveness [4]. Studies increasingly show that positive psychological constructs are significantly associated with patients’ quality of life, mental health and satisfaction with life, particularly among cancer patients [2, 5–8]. Thus, enhancing these constructs is crucial for the well-being of cancer patients.

Cancer is the leading cause of death globally, and according to World Health Organization (WHO) reports, it is the first or second leading cause of death among those younger than 70 years in most countries [9]. Cancer incidence and mortality rates have increased rapidly across countries of all income levels. In addition, cancer diagnosis and subsequent treatment are highly stressful for patients, which often leads to negative emotions and deterioration in mental health [10]. Studies show that cancer patients with similar disease severity and treatment status often display significantly different levels of psychological stress, likely due to disparities in terms of hope, resilience, optimism and self-efficacy [5–8]. Depression and anxiety are the most common psychological distress among cancer patients [11]. A growing number of studies have reported that positive psychological resources, as protective factors, can help cancer patients adjust and manage disease, thereby effectively attenuating psychological distress and mental problems [12, 13]. For instance, Yang et al. found that the integrated effects of hope, resilience and optimism were significantly and negatively associated with depression and anxiety among patients with cancer [14]. Therefore, evaluating the positive psychological constructs can help assess the mental health of cancer patients during treatment.

However, the current evaluation scales are based on participants with depression and anxiety or the general population [15, 16]. For instance, the Connor Davidson Resilience Scale (CD-RISC) was developed based on a sample with anxiety symptoms [17]. In addition, the Snyder Hope Scale was broadly adapted to measure the level of hope among heathy individuals [18, 19]. Some aspects of these state-like variables and integration processes in cancer patients are significantly different from those in other populations due to the complex treatment and potential fatality in the former [20–22]. Ye et al. surmised that cancer patients need to learn new skills to self-manage fatigue, pain, nausea, and constant negative emotion concerning death throughout the process of treatment [21]. Therefore, it is crucial to develop scales specific to cancer patients to gauge their mental health with greater reliability. Luthans et al. [23] proposed the concept of psychological capital (PsyCap), which consists of hope, resilience, self-efficacy and optimism and shows a relatively stronger relationship to performance and job satisfaction than any of the individual facets. The aim of our study was to develop and validate a PsyCap questionnaire for cancer patients to assess their mental state and the ability to self-manage and “bounce back” after cancer diagnosis and treatment.

## Methods

### Survey participants

The survey was administered to cancer patients across five cities in northeast China between September 2020 and February 2021. The inclusion criteria for patients were as follows: 1) cancer diagnosis, 2) awareness of the disease, 3) fluency in Chinese, and 4) 18 years or older. Patients with other severe diseases (such as cardiovascular disease, history of psychiatric problems, or cognitive and intellectual disorders) were excluded. The participants were interviewed to obtain all relevant information. The study was approved by the Ethics Committee on Human Experimentation of China Medical University, and all participants provided written consent prior to the survey.

### Measurements

#### Depression

Depression was evaluated using the Chinese version of the self-reported Patient Health Questionnaire-9 (PHQ-9) [24] over a period of two weeks. The PHQ-9 includes nine items related to anhedonia, sadness, sleep, fatigue, appetite, feelings of worthlessness, concentration, motor skills and death. Higher scores indicate higher levels of depression. The Cronbach’s alpha for the PHQ-9 is 0.869.

#### Anxiety

Anxiety was assessed using the Chinese version of the self-reported Generalized Anxiety Disorder-7 (GAD-7) [25] over two weeks. The GAD-7 contains seven items related to nervousness, control, worry, relaxation, restlessness, irritability and fear. Higher scores indicate higher levels of anxiety. The Cronbach’s alpha for the GAD-7 is 0.895.

### Statistical methods

#### Item generation

The preliminary questionnaire was developed on the basis of a literature review and semi-structured and in-depth interviews with 30 cancer patients. Following discussion with an expert panel of psycho-oncologists and grouping/excluding any similar or ambiguous items, a 64-item questionnaire was drafted. Each item was scored on the basis of a five-point Likert scale that varies from “1=very strongly disagree” to “5=very strongly agree”. A pilot survey was then carried out on 202 cancer patients to assess the validity and clarity of the questionnaire, and the number of items was reduced to 44.

#### Item reduction and scale development

A total of 505 cancer patients were recruited on the basis of the inclusion and exclusion criteria, and their demographic and clinical data were collected. The intercorrelation between the items was calculated based on the data generated from the survey. Low interrelation items (*r* < 0.1) were excluded, and the highly interrelated items (*r* > 0.7) were analysed further. The least clinically relevant item was excluded due to high association with the same underlying dimension theoretically [26].

#### Construct validity

Exploratory factor analysis (EFA) was used to assess construct validity to identify the key components. The suitability of EFA was assessed by Bartlett’s test, Kaiser-Meyer-Olkin (KMO) measure and eigenvalue cut-off value > 1. The items with total correlation < 0.3, factor loading < 0.5 on one factor, communality < 0.4 and loaded into two factors were excluded [27]. Structural equation modelling (SEM) was used to perform confirmatory factor analysis (CFA). After optimizing all parameters, the *χ*^2^ test and measures for good of fit were reviewed [28, 29].

#### Reliability

Cronbach’s alpha was used to evaluate the internal consistency for the total scale [29]. The test-retest ability among 100 cancer patients with a four-week interval between the tests was measured by Spearman’s correlation analysis. The criterion validity of the PCQ-C, including depression and anxiety, was evaluated based on the questionnaires completed by 227 breast cancer patients.

## Results

### Participants

A total of 505 cancer patients completed the questionnaires, and five were subsequently excluded on account of considerable missing data (> 30%). Therefore, the effective response rate was 99%. The mean age of the participants was 58.03 years (SD: 12.35), and 56.2% of the participants were females and 43.8% males. Approximately 76% of the patients were married/cohabiting. Furthermore, 76.2% of the patients had a high school level or lower educational level, and 82.8% were employed. Approximately 46.6% of the participants had a monthly income over 3000 yuan. The proportions of patients with lung cancer, breast cancer and haematological cancers were 20.8, 15.2 and 14.8%, respectively (Table 1).

**Table 1:** Demographic characteristics of the participants (*n* = 500)

Variables		N (%)
Age	Mean ± SD	58.03 ± 12.35
Gender	Male	219 (43.8)
	Female	281 (56.2)
BMI	Mean ± SD	23.55 ± 4.72
Marital status	Married/cohabiting	383 (76.6)
	Single/separated/divorced/widowed	74 (14.8)
	Missing	43 (8.6)
Education	High school or below	363 (72.6)
	Some college	68 (13.6)
	College or above	41 (8.7)
	Missing	28 (5.6)
Employment status	Employed	414 (82.8)
	Unemployed/temporary workers	62 (12.4)
	Missing	24 (4.8)
Monthly income	< 3000	233 (46.6)
	3000–5000	156 (31.2)
	≥ 5000	79 (15.8)
	Missing	32 (6.4)
Cancer type	Hematologic	74 (14.8)
	Colon	40 (8.0)
	Lung	104 (20.8)
	Cervical	24 (4.8)
	Breast	76 (15.2)
	Esophagus	17 (3.4)
	Head and neck	24 (4.8)
	Gastric	22 (4.4)
	Other	37 (7.4)
	Missing	82 (16.4)

### Construct validity

#### Exploratory factor analysis

Five items were deleted owing to item-total correlation < 0.3. Therefore, all 39 items were analysed using EFA, which initially generated six factors (eigenvalue > 1) with a total explained variance of 56.26%. (KMO = 0.951, Bartlett’s test of sphericity *χ*^2^ = 9662.41, df = 741, *P* < 0.001). However, the fit was poor, and 10 items were excluded due to low factor loading (< 0.5) and cross-loading on two factors. The remaining 29 items were analysed further, and 5 were extracted, explaining 57.21% of the variance (KMO = 0.941, Bartlett’s test of sphericity *χ*^2^ = 6622.52, df = 406, *P* < 0.001). One factor (two items) was removed, as the explained variance of 4.59% (eigenvalue of 1.36) was lower than 5% [27]. Three items were omitted due to insignificant factor loading (< 0.5) and low community (< 0.4). After a final round of EFA on the remaining 24 items, 4 were produced and explained 56.72% of the variance, and each factor was over 10% of the explained variance (KMO = 0.925, Bartlett’s test of sphericity *χ*^2^ = 5382.19, df = 276, *P* < 0.001) (Table 2).

**Table 2:** Results of the principal components analysis of 24 items

Items	Factor 1	Factor 2	Factor 3	Factor 4
I can still experience the joys of life after being diagnosed with cancer	0.575			
Do something special for myself to make life better	0.652			
Help other patients cope with cancer and treatment	0.622			
Divert my attention from the disease by focusing on other important things in life	0.769			
Find a way to help myself through this difficult time	0.771			
I can overcome physical distress or relieve fatigue by doing something	0.762			
I treat disease with a positive attitude		0.652		
I never give up even if the chance of cure is low		0.729		
My faith helps me through my illness		0.675		
I can give and receive love and care from others		0.632		
I should perform positive actions to navigate through the disease		0.708		
I believe that we can fight diseases as long as we work hard		0.681		
I like to challenge myself with new and difficult things			0.716	
I still think I’m a very energetic person when sick			0.715	
My daily life is full of things that interest me			0.715	
I have a tenacious personality even after the cancer diagnosis			0.669	
I can concentrate and think clearly under the pressure of illness			0.647	
I can make unusual or difficult decisions			0.602	
If I think I am going to fail, it is going to happen				0.584
The effect of disease treatment rarely follows the trend I expect				0.583
I often worry about my health				0.706
I hardly expect good things to happen to me				0.556
I feel tired most of the time				0.666
I feel very lonely or helpless after cancer diagnosis				0.633
Eigenvalue	3.826	3.693	3.641	2.452
% of variance	15.944	15.387	15.170	10.218

Factor 1, self-efficacy; factor 2, hope; factor 3, resilience; factor 4, optimism

Based on the EFA results, the first factor contained six items and was identified as hope concerning the expectations of a cancer cure and confidence in fighting the disease. The second factor was defined as self-efficacy and contained six items related to the ability to cope with treatment-related side effects. Six items loaded on the third factor, which was identified as resilience, adaptability and past achievements. Finally, six items described optimism related to positive attitudes. The eigenvalues of these four factors were 3.826, 3.693, 3.641 and 2.452, respectively.

#### Confirmatory factor analysis

**Fig. 1:**
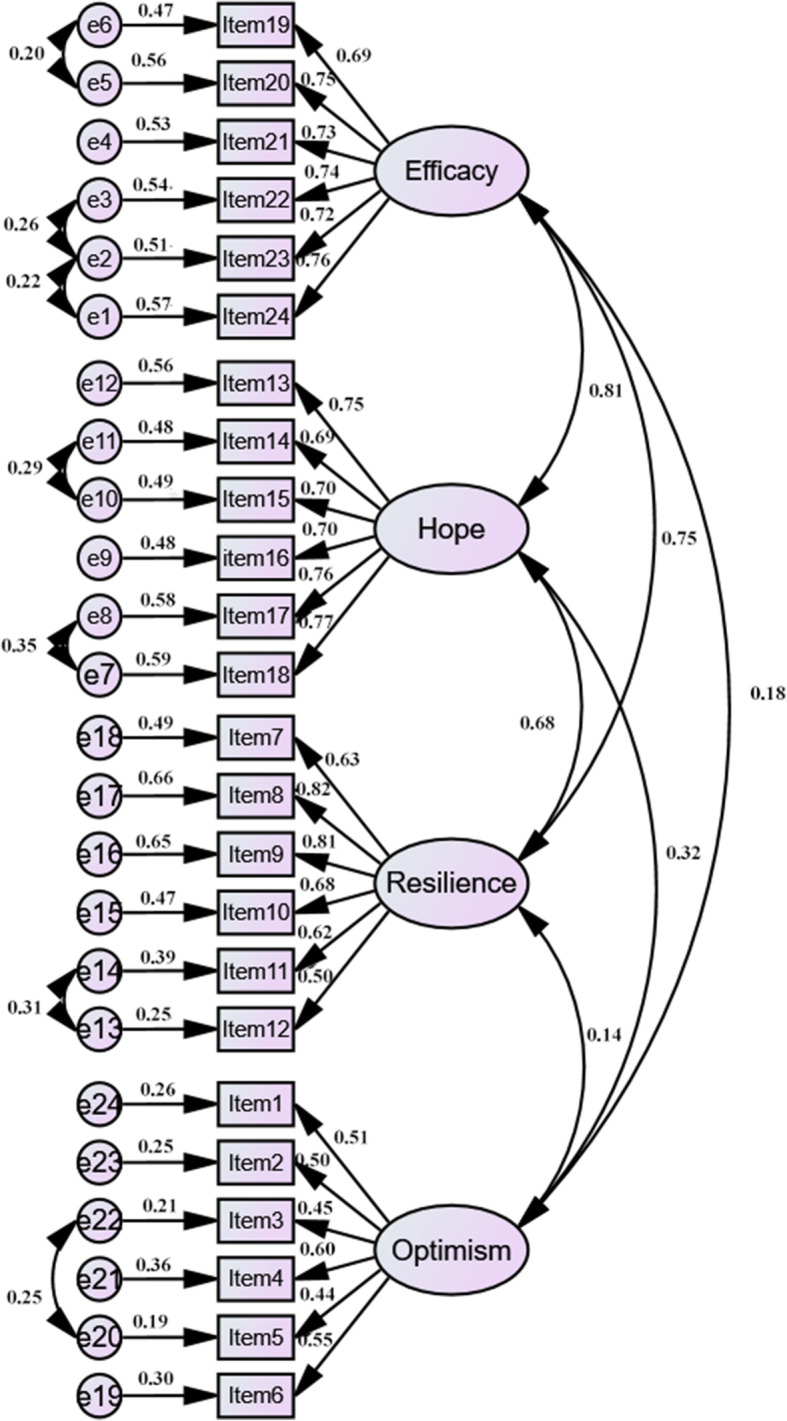
Final CFA model of the PCQ-C

The goodness-of-fit of the four-factor structure of the PCQ-C tool was determined using AMOS version 17.0, which revealed excellent fit indices (χ^2^/df = 2.072, GFI = 0.925, RMSEA = 0.046, CFI = 0.951, NFI = 0.910, TLI = 0.943, AGFI = 0.906 and IFI = 0.951) [30–32]. According to the modification indices, several paths of covariance between items and error were added to enhance the fit indices (Fig. 1).

### Internal consistency, test-retest reliability and criterion-related validity

#### Internal consistency

The Cronbach’s alpha coefficient (*α*) for the total scale was 0.886, which is considerably over the recommended index of 0.70 [32]. The internal consistency and correlation value of corrected items to the total correlation of items to their loading subscale are shown in Table 3.

**Table 3:** Item to total correlation and Cronbach’s alpha for the scale

Scale/items	Corrected item to total correlation	Cronbach’s alpha
Self-efficacy		0.880
19. I can still experience the joys of life after being diagnosed with cancer	0.763^**^	
20. Do something special for myself to make life better	0.758^**^	
21. Help other patients cope with cancer and treatment	0.708^**^	
22. Divert my attention from the disease by focusing on other important things in life	0.652^**^	
23. Find a way to help myself through this difficult time	0.648^**^	
24. I can overcome physical distress or relieve fatigue by doing something	0.712^**^	
Hope		0.879
13.I treat disease with a positive attitude	0.782^**^	
14.I never give up even if the chance of cure is low	0.598^**^	
15. My faith helps me through my illness	0.751^**^	
16. I can give and receive love and care from others	0.639^**^	
17. I should perform positive actions to navigate through the disease	0.720^**^	
18. I believe that we can fight diseases as long as we work hard	0.720^**^	
Resilience		
7. I like to challenge myself with new and difficult things	0.456^**^	
8. I still think I’m a very energetic person when sick	0.735^**^	0.839
9. My daily life is full of things that interest me	0.676^**^	
10. I have a tenacious personality even after the cancer diagnosis	0.602^**^	
11. I can concentrate and think clearly under the pressure of illness	0.653^**^	
12. I can make unusual or difficult decisions	0.576^**^	
Optimism		
1. If I think I am going to fail, it is going to happen	0.311^**^	
2. The effect of disease treatment rarely follows the trend I expect	0.312^**^	0.696
3. I often worry about my health	0.337^**^	
4. I hardly expect good things to happen to me	0.416^**^	
5. I feel tired most of the time	0.306^**^	
6. I feel very lonely or helpless after cancer diagnosis	0.450^**^	
PsyCap-C		0.886

^**^ *P* < 0.01

PsyCap-C, Psychological Capital to Cancer

#### Test-retest reliability

Test-retest reliability testing of the scale was conducted on the data collected from 120 cancer patients with a four-week interval between the tests. The response rate was 83.3% (100 participants) since twenty patients rejected further investigation. The mean age of the participants was 57.04 years (SD: 12.32); 55% were females and 45% were males. The mean PsyCap-C scores at the first and second assessment occasions (T1 and T2) were 3.81 (SD = 0.51) and 3.64 (SD = 0.46), respectively. There was a significant correlation between the two periods (PsyCap-C: *r* = 0.825, *P* < 0.001; self-efficacy: *r* = 0.824, *P* < 0.001; hope: *r* = 0.836, *P* < 0.001; resilience: *r* = 0.811, *P* < 0.001; optimism: *r* = 0.807, *P* < 0.001), and Cronbach’s alpha coefficient (*α*) was adequate at both time points (Table 4).

**Table 4:** Cronbach’s alpha and test-retest reliability at the first and second assessment occasions

Scale	Cronbach’s alpha T1	Cronbach’s alpha T2	Test-retest reliability
Self-efficacy	0.880	0.894	0.824^***^
Hope	0.879	0.894	0.836^***^
Resilience	0.839	0.841	0.811^***^
Optimism	0.696	0.754	0.807^***^
PsyCap-C	0.886	0.911	0.825^***^

^***^ *P* < 0.001

T1, time 1; T2, time 2

#### Criterion-related validity

Among 227 patients with breast cancer, the mean scores of depression and anxiety were 6.83 (SD = 4.87) and 5.68 (SD = 4.23), respectively. PsyCap-C and the four individual dimensions showed a significant association with depression (PsyCap-C: *r* = − 0.631, *P* < 0.01; self-efficacy: *r* = − 0.536, *P* < 0.01; hope: *r* = − 0.525, *P* < 0.01; resilience: *r* = − 0.479, *P* < 0.01; optimism: *r* = − 0.400, *P* < 0.01) and anxiety (PsyCap-C: *r* = − 0.601, *P* < 0.01; self-efficacy: *r* = − 0.502, *P* < 0.01; hope: *r* = − 0.477, *P* < 0.01; resilience: *r* = − 0.449, *P* < 0.01; optimism: *r* = − 0.416, *P* < 0.01).

## Discussion

Evaluation of PsyCap consisting of hope, self-efficacy, optimism and resilience can improve the quality of life of cancer patients, help them cope with mental problems (depression and anxiety), and even relieve cancer-related fatigue [5–8, 33]. However, the scales used thus far are either generic PsyCap tools such as the PCQ [23], which do not represent the unique psychological condition of cancer patients, or one-dimensional scales such as the Cancer Behaviors Inventory (CBI) [22], which neglects all psychological propensities apart from coping ability. Given that PsyCap may have a relatively stronger association with positive effects in cancer patients compared to the four individual facets, we developed a questionnaire based on both patients’ and experts’ views of the disorders. This is the first questionnaire that was developed to assess PsyCap specifically in cancer patients. The items for the scale were selected on the basis of a literature review and semi-structured patient interviews wherein they expressed their views regarding the disease [34]. Subsequently, psycho-oncology experts reviewed and assessed the selected items, which were then validated on 500 cancer patients using EFA and CFA, which indicated good internal consistency and test-retest reliability on the PCQ-C. Finally, the PCQ-C has 24 items, and a four-factor structure is identified.

The final version of the PCQ-C consists of four domains: hope (six items), self-efficacy (six items), resilience (six items) and optimism (six items). Hope is a positive expectation about achieving a possible and significantly good future in fighting cancer [35]. Self-efficacy is defined as a positive belief of individual competence to deal with treatment-related side effects or achieve desired goals in the face of cancer [36]. Resilience is expressed in an individual’s capacity to successfully recover and maintain their mental health in the context of disease events [37]. Optimism is considered the degree of general expectation that positive outcomes will happen rather than bad things in the face of cancer diagnosis and treatment [38, 39]. In our study, PsyCap-C and the four individual dimensions were significantly associated with depression and anxiety, which suggested that the scale had good construct validity.

### Clinical implications

Our study has developed the first objective measure of PsyCap specifically for cancer patients. We hope that it can be applied in the clinical setting to monitor PsyCap as part of cancer care and in psychological interventions as a self-report outcome measure.

### Study limitations

There were several limitations in the current study. First, the cross-sectional design precluded the verification of developmental stability or change in PsyCap for the sample. Therefore, the time-dependent changes in the sensitivity of the PCQ-C will have to be assessed in future studies. Second, although the PCQ-C was based on PsyCap theory, which mainly originated from studies in the West, we developed and validated this scale among Chinese cancer patients. Therefore, the applicability of the PCQ-C should be tested across different ethnicities. Third, our study lacked convergent validity for the scale and did not compare the PCQ-C with other scales. The self-reported data in our study might also have resulted in bias. Finally, items in the optimism dimension presented low item-total correlations compared to other items and will have to be revised.

## Conclusions

The PCQ-C is a reliable tool to objectively measure PsyCap among cancer patients based on self-efficacy, hope, resilience and optimism.

## Data Availability

The dataset in this study is available from the corresponding author on reasonable request.

## Abbreviations

PsyCap: Psychological Capital
PCQ-C: Psychological Capital Questionnaire for cancer patients
WHO: World Health Organization
CD-RISC: Connor Davidson Resilience Scale
PHQ-9: Patient Health Questionnaire-9
GAD-7: Generalized Anxiety Disorder-7
EFA: Exploratory factor Analysis
KMO: Kaiser-Meyer-Olkin
CFA: Confirmatory Factor Analysis
